# The use of surfactant-filled mesoporous silica as an immobilising medium for a fluorescence lifetime pH indicator, providing long-term calibration stability†

**DOI:** 10.1039/c9ra07374d

**Published:** 2019-11-14

**Authors:** C. Totland, P. J. Thomas, B. Holst, N. Akhtar, J. Hovdenes, T. Skodvin

**Affiliations:** Department of Chemistry, University of Bergen Allégaten 41 5020 Bergen Norway christian.totland@ngi.no; Norwegian Research Centre Fantoftvegen 38 Bergen Norway; Department of Physics and Technology, University of Bergen Allégaten 55 5020 Bergen Norway; Aanderaa Data Instruments AS Sanddalsringen 5b N-5225 Nesttun Norway

## Abstract

Surfactant filled mesoporous silica is applied as a matrix for immobilizing the fluorescence lifetime pH-indicator acridine. We demonstrate that this type of encapsulation provides a stable and uniform chemical environment for the indicator and has good proton transport properties leading to rapid pH response times. Furthermore, the immobilising medium effectively prevents leaching of the indicator, facilitates high long-term stability and does not influence the pH sensing-range of the indicator.

## Abstract 2

A material support is proposed which grants long-term stability for fluorescence lifetime pH measurements due to a homogeneous chemical microenvironment.

## Graphical abstract

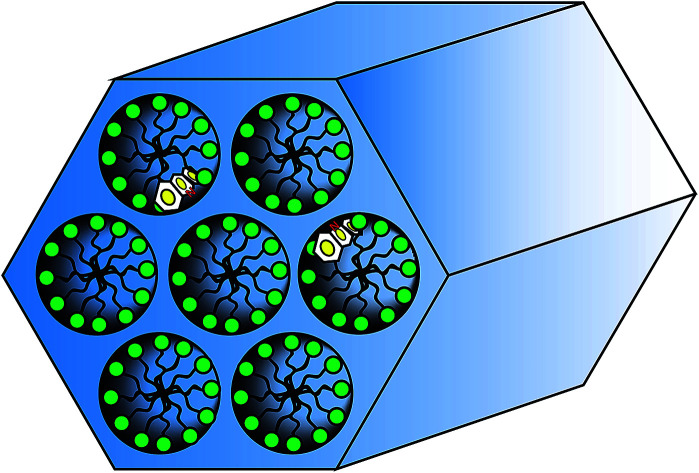

The most common pH sensor is the electrochemical sensor. Although electrochemical pH sensors are used for a range of analytical purposes, they have limitations that make them unsuitable for some applications. One obvious application is physiological pH measurements, where *e.g.* pH is a potential tool for *in situ* diagnostics.^1^ Moreover, electrochemical pH sensors require frequent calibration and are therefore not ideal for environmental applications, such as for water quality monitoring in rivers, the ocean, fish farms or pipelines, where long-term continuous operation is desired and frequent field maintenance is a drawback. Standalone optical sensors comprising membrane-immobilized indicators are attracting an increasing amount of attention for environmental applications due to their higher stability, small size, low power consumption and moderate cost.^2,3^

Such optical sensors often incorporate fluorophores as pH indicators, where both the fluorescence intensity and lifetime can depend on pH over a limited pH range of about 2–3 pH units about the p*K*_a_ of the indicator.^4^ Although such a limited pH sensing range excludes a number of applications, it is sufficient for most environmental and physiological purposes. A drawback with fluorescence intensity-based pH monitoring is that the intensity of the emitted light will also be sensitive to other variables that are difficult to bypass completely, such as leaching of the indicator, photobleaching and variations in the probe light intensity. Fluorescence lifetime (FL), on the other hand, is an intrinsic property of the indicator and will be independent of these durability concerns, providing a sufficient signal-to-noise ratio is maintained.

The possibility of a more reliable pH sensor based on FL has been proposed frequently in literature, both as standalone sensors^5,6^ and as physiological pH probes when combined with imaging techniques.^1,7^ However, a source of uncertainty for such measurements is the dependence of FL on the chemical environment of the indicator, potentially causing instability and uncertainty. For example, in the complicated environment of a living cell, interactions between fluorophores and cell constituents lead to variable FL and unreliable pH measurements.^7^ The FL of an indicator will depend on solvation dynamics, molecular rotation, polarity of the environment, Förster resonance energy transfer and charge transfer, among other things.^8^ Hence, the FL depends not only on pH, but also on the environment where the indicator is located.

In the case of standalone sensors, the properties of the film that immobilizes the indicator is therefore of crucial importance for the sensor performance.^5,9^ The non-uniform nature of most polymer-based films or coatings leads to additional instability, as slow migration of the indicator between different environments may occur over time. Changes in pH can potentially initiate such migrations.^10,11^

Sol–gel silica is another common support material used to immobilize indicators.^12–15^ However, potential indicators are often non-polar, which restricts the loading capacity in porous silica. Moreover, in absence of covalent bonds, the indicator may interact weakly with the pore surface, which makes leaching an issue for long-term use.

The intact surfactant template of mesoporous silica has been proposed previously as host for chemo-sensing molecules in general.^16,17^ Here, we present a similar way to encapsulate a FL indicator in order to keep it in a more constant chemical environment. This removes many reliability concerns and allows more accurate external pH calibration. Moreover, non-polar indicators will dissolve in the surfactant non-polar phase, which drastically increases the loading capacity of the material. When used as part of a standalone sensor, the encapsulation could protect the indicator from the influence of other compounds within the membrane that may otherwise influence the pH response characteristics.

Acridine is among the few organic fluorophores with a large lifetime variation with pH.^5,18^ Hence, this fluorophore was chosen for the study. The protonated and deprotonated forms of this amine have fluorescence lifetimes of 10 and 31 ns, respectively.

In the synthesis of the silica, acridine is dissolved in the surfactant (tetradecyltrimethylammonium bromide, TTAB) phase (1 : 10 mole ratio) prior to addition of the silicon alkoxide. The TTAB phase is kept intact in the final powder material. Transmission electron microscopy (TEM) and ^29^Si magic angle spinning (MAS) NMR was used to characterize the material. The NMR data gives a Q3/Q4 ratio of 0.52, which is similar to *e.g.* MCM-41 (ESI†).^19^ For comparison, the Q3/Q4 ratios of *e.g.* MCM-48 (cubic) and MCM-50 (lamellar) are expected around 1.1 and 1.4, respectively.^19^ However, the transmission electron micrographs reveal a more amorphous pore structure (ESI†). A more spherical pore structure is expected as TTAB does not form a liquid crystalline phase similar to that of cetyltrimethylammonium bromide (CTAB) used as a template for MCM41, although the inclusion of acridine may favor a more elongated pore structure.

Fig. 1 compares the pH dependency of the fluorescence lifetime of free acridine and acridine immobilized in the surfactant phase of the mesoporous silica material. The data shows that the pH sensing range of the indicator is effectively unchanged by incorporation in the material. A very high acridine loading was achieved, with 3.7 wt% acridine in the dry powder.

**Fig. 1:**
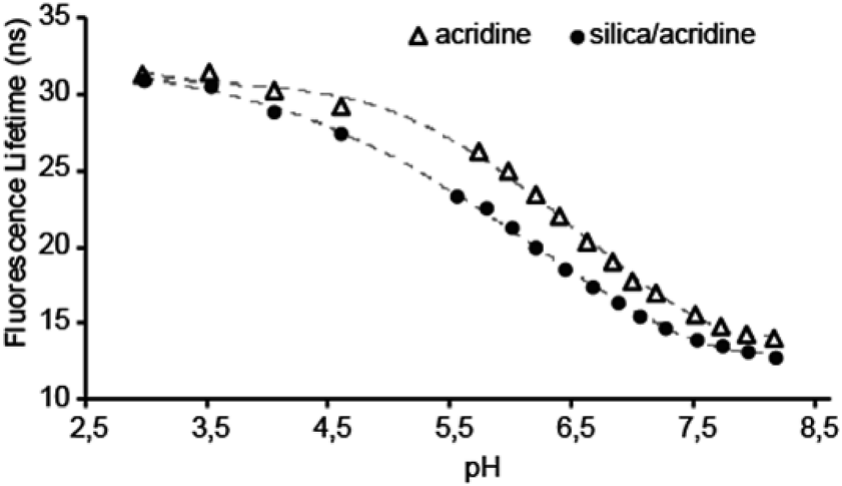
Fluorescence lifetime *versus* pH for acridine (Δ) and acridine in the surfactant phase of the silica (●). Measurements are done in 100 mM phosphate buffers (NaH_2_PO_4_/Na_2_HPO_4_ from pH 5.8–8, and Na_3_PO_4_/BH_3_O_3_ below pH 5.8).

Consequently, the high fluorescence intensity enables a low uncertainty on the lifetime measurements. This particular system will be most accurate in the pH range between pH 5.3 to 7.3, where FL and pH are linearly dependent and a change in pH of 0.1 gives a change in FL of 0.5 ns. The total change in FL between pH 3 and 8 of about 20 ns for acridine meaning that it greatly outperforms other known FL indicators in this pH range, that typically have a maximum change in FL of 1–2 ns.^8^

The response in FL to changes in pH occurs quicker than the time it takes to set up and run a measurement, *i.e.* less than a minute. This observation indicates rapid transport of protons through the material.

Due to the high sensitivity of FL to changes in the local chemical environment surrounding the indicator, the most important parameter for a FL support material is long-term stability. Moreover, this stability may change depending on the dispersing solution. The material used to obtain the data in Fig. 2 was stored in either distilled water (Milli-Q), 3.5% NaCl, 100 mM pH 5.8 and pH 8 buffers, and extracted and re-dispersed in a new solution prior to every measured data point. Fig. 2 shows that the measured fluorescence lifetime of the material was stable over one month in 100 mM pH 5.8 and pH 8 buffers, as well as in 3.5% NaCl. However, in Milli-Q water, the FL declines steadily. When dissolved in Milli-Q water without the immobilizing material, the FL of acridine is unchanged with time: a fresh concentrated aqueous solution (0.25 mM) of acridine was compared to one that had been stored for about two years. The FLs were identical within uncertainty (old = 15.22 ns; new = 15.37 ns). Hence, the observed decline in FL is related to the material. Since the material is analyzed in dispersion, electrolytes may facilitate greater material stability due to increased aggregation behavior. However, the reason for the observed instability in Milli-Q water is not fully understood.

**Fig. 2:**
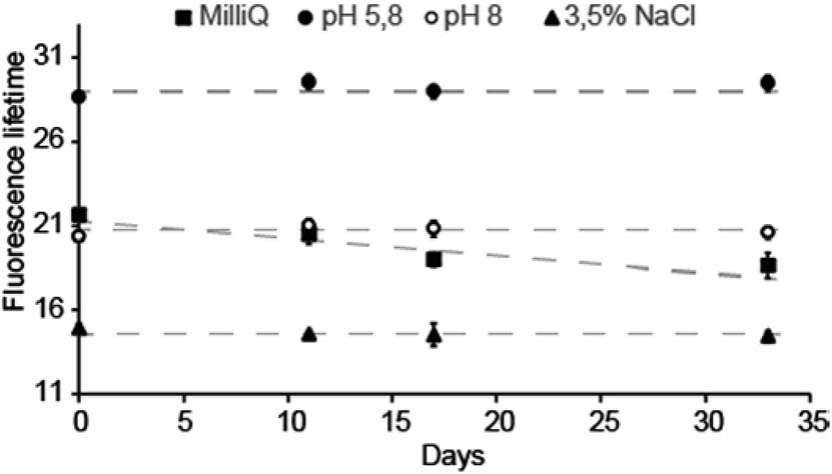
Fluorescence lifetime long-term stability of the of the acridine-mesoporous silica material in four different solutions; Milli-Q water, 3.5% NaCl and 100 mM phosphate buffers at pH 5.8 and 8.0. The data points are averages of five consecutive measurements, and the error bars are 2× the standard deviation of these.

Note that the lower FLs measured in NaCl (Fig. 2) are due to the presence of chloride; a well-known issue with acridine whereby the fluorescence is quenched by chloride.^20^ The transport of chloride anions was investigated by adding NaCl solution to the dispersion to give a final NaCl concentration of 100 mM. As with protons, the effect of the chloride ions was observed instantly, indicating that cations and anions transport are equally effective in the material. The chloride caused the FL to drop instantly to around 10 ns when NaCl was added to the dispersion, with marginal dependence on pH (between pH 5.8 and 8.0). This is similar to free acridine in solution,^5^ and reflects that the dynamic quenching occurs for the protonated acridine only. Fluorescence is therefore only observed from the 10 ns lifetime neutral acridine species. Without use of chloride protective membranes, this system will only function as a FL pH indicator in environments with [Cl^−^] below 20 mM.^5^ This excludes use in extracellular environments ([Cl^−^] = 100 mM), but allows intracellular ([Cl^−^] = 4 mM) and blood ([Cl^−^] = 10 mM) applications. For sensor applications, the material can be further protected to prevent Cl^−^ quenching, *e.g.* by using a film of semipermeable Nafion.^5^ Note that acridine is not quenched by other halogen ions and is therefore not affected by the bromide present in the TTAB phase.^20^

The amount of acridine in the final samples was about 37 mg g^−1^ material, which gives a TTAB : acridine mole ratio of 12 : 1. This is slightly less than the 10 : 1 ratio of the initial TTAB : acridine mixture. Fig. 3 shows that tumbling a small amount of powder in various solutions causes between 0.3–0.8% of the original acridine present to leach within 15 days. In 3.5% NaCl and pH 8 buffer, no further leaching is detected following the washing procedure in Milli-Q water. In Milli-Q water, the leaching seems to proceed at a rate of about 0.2% per month. When placed in a slightly acidic buffer of pH 5.8, about 0.2% additional acridine leaches, after which no further leaching is detected the following eight days of continued tumbling in the buffer. In fact, the leaching was found to be inversely proportional to pH, where the percentage of remaining acridine after 5 days (%_acr_) can be estimated from: %_acr_ = 0.13 × pH + 98.4 (ESI†). Still, at any relevant pH value, the leaching will be insignificant regarding fluorescence sensor applications.

**Fig. 3:**
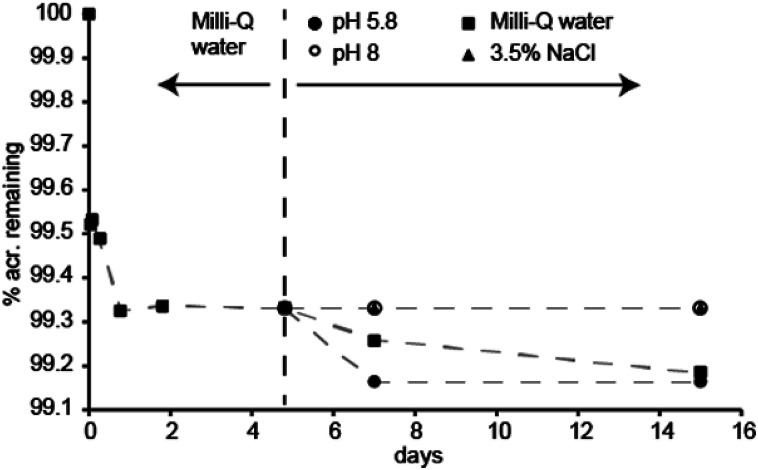
Leaching of acridine from the material. The material was tumbled in Milli-Q water for 5 days as part of the washing procedure. Following this, the material was divided in four solutions; Milli-Q water, 3.5% NaCl, 100 mM phosphate buffers of pH 5.8 and 8.0, and tumbled for another ten days.

The location of acridine inside the material was evaluated by MAS ^1^H NMR. Fig. 4 indicates the changes in chemical shift of the resonances corresponding to protons a–d when acridine is incorporated into the TTAB phase of the mesoporous silica. The most notable changes are a large upfield shift (−0.26 ppm) for proton ‘a’ and a similar downfield shift (+0.26 ppm) for proton ‘b’. This can be rationalized by an interaction between the nitrogen and hydroxyls at the surface. This will alter the bond polarization about the nitrogen which will mostly affect the nuclear shielding of the proton in the same ring (proton a) as well as those in close proximity to the nitrogen (proton b). Acridine is therefore located in the palisade region of the TTAB phase, with the nitrogen pointing towards the pore walls (Fig. 4). This makes the nitrogen accessible to protons diffusing between the pore walls and the TTAB head groups.

**Fig. 4:**
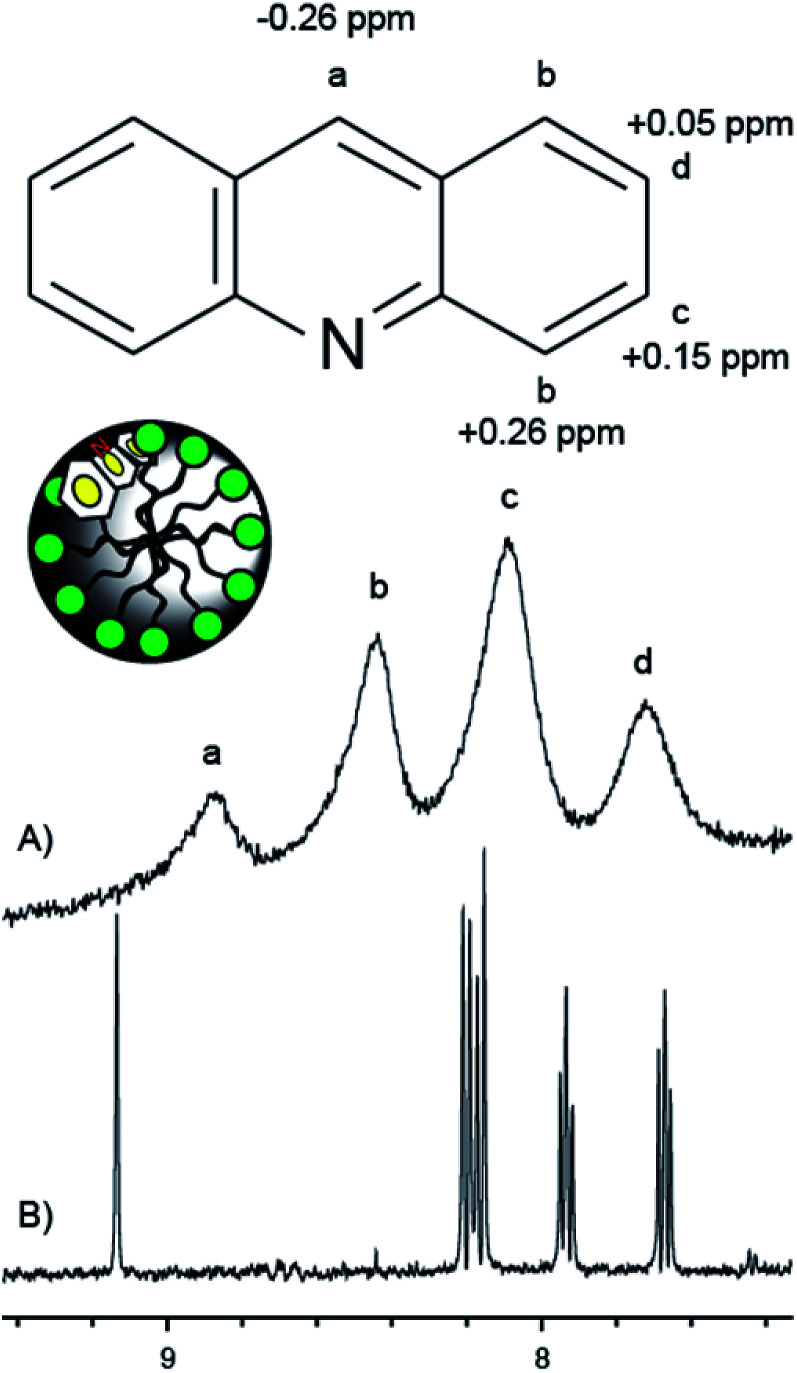
^1^H NMR spectra of acridine (500 MHz) in solution and incorporated in the MCM-41/CTAB material: (A) MAS spectrum (10 kHz MAS) of powder wetted with D_2_O. (B) Solution ^1^H NMR spectrum. Both spectra are recorded with water suppression and using DSS as chemical shift reference. The changes in chemical shift upon incorporation in the silica material is indicated in the molecular structure of acridine for protons a–d. The location of acridine inside a pore is schematically illustrated.

For real applications, the material may need further refinement, such as incorporation into a film or substrate in the case of standalone sensors, or addition of target-specific substituents in the case of physiological pH measurements. An advantage with the system presented here is that the indicator is protected in the interior of the material, and changes to the material exterior will not affect its chemical environment. This particular material can function in several physiological environments, as well as in seawater applications following additional protection from chloride by *e.g.* Nafion. The material may further be an interesting candidate as host material for other types of molecules, both regarding indicators for pH and other sensor applications, and as a carrier for drugs. One potential application in this respect is for example photodynamic therapy for cancer treatment, where the hydrophobicity of the photosensitizers limits the amount and effect of the light therapy in cancer cells.^21^

## Conflicts of interest

There are no conflicts to declare.

## Supplementary Material

RA-009-C9RA07374D-s001
