# Supplementary material for: The use of surfactant-filled mesoporous silica as an immobilising medium for a fluorescence lifetime pH indicator, providing long-term calibration stability
Source: RSC Adv. 2019 Nov 14;9(64):37241–4. doi: 10.1039/c9ra07374d (PMC9075546; doi:10.1039/c9ra07374d)
Supplement: RA-009-C9RA07374D-s001 [file RA-009-C9RA07374D-s001.pdf]

Electronic Supplementary Material (ESI) for RSC Advances.
This journal is © The Royal Society of Chemistry 2019

Supporting Information

The use of surfactant-filled mesoporous silica as an immobilising medium for a fluorescence lifetime pH indicator, providing long-term calibration stability

Christian Totland*[a,b], Peter J Thomas[c], Bodil Holst[d], Naureen Akhtar[d], Jostein Hovdenes[e] & Tore Skodvin[a]

[a]Department of Chemistry, University of Bergen
[b]Norwegian Geotechnical Institute
[c]NORCE Norwegian Research Center AS, Fantoftvegen 38, 5072 Bergen, Norway
[d]Department of Physics and Technology, University of Bergen
[e]Aanderaa – A Xylem Brand

This pdf includes:

- **Materials and Methods**
- **^{29}Si MAS NMR and TEM characterization data**
- **Additional leaching data**
- **Fluorescence spectra**

Materials and Methods

Acridine (97%), tetraethoxysilane (TEOS, 98 %) and tetradecyltrimethylammonium bromide (TTAB) was obtained from Sigma Aldrich. A mesoporous silica was synthesized by mixing 2.3 mmoles of TTAB and 0.23 mmoles of acridine in 16.7 mL water, which was gently heated to dissolve the powders completely. After cooling to room temperature, 25.3 mL EtOH and 4.4 mL NH_3 were added and the solution stirred for 15 minutes. Then, 1.66 mL TEOS was added and the solution stirred for an additional 2 hours. The powder was washed four times by shaking with 40 mL water followed by centrifugation to remove the water, and then left to tumble with additional 40 mL of water for five days. The powder was finally dried at 50° overnight. Acridine then remains in the TTAB phase within the mesoporous network.

100mM phosphate buffers are used: NaH_2PO_4/Na_2HPO_4 from pH 5.8-8, and Na_3PO_4/BH_3O_3 below pH 5.8).

Soild-State NMR spectroscopy. The NMR experiments were obtained using a Bruker AVANCE III 500 MHz instrument. The instrument is equipped with a magic angle spinning (MAS) probe for 4 mm rotors. Experiments were carried out at a sample temperature of 298 K, with sample spinning rate of 10 kHz. For ^{1}H MAS NMR, water signal suppression was applied by pre-saturation pulses. ^{29}Si MAS NMR spectra were recorded using inverse-gated ^{1}H decoupling, 1000 transients and a relaxation delay of 120 seconds between each transient. Prior to packing the MAS rotors, the powdered samples were dispersed in D_2O, followed by sonication for 5 minutes to create a finer dispersion. The excess D_2O was removed by centrifugation at 18000 rpm, and the moist powder was packed into a rotor. D_2O was primarily added to increase the mobility of functionalized surface species of trapped acridine, in order to obtain a better resolution.

Leaching. The fresh material was tumbled in water for five days, and leaching was tested every hour for the five first hours. 1 mL of the suspension was extracted, centrifuged, and the

supernatant analyzed with UV spectroscopy (at 354 nm). Exact concentrations of acridine were determined by comparison with a calibration curve for UV absorption.

Then, samples were extracted and analyzed daily the following five days. The water needed to be replaced after the first hour as the amount of leached acridine approached the solubility limit in water of 0.25 mM. Following the five days of tumbling in water and drying of the material, four new samples were prepared by mixing a weighed amount (about 0.05 g) of the same powder / material in 5 mL of either milli-q water, 3.5% NaCl, 100 mM pH 8 phosphate buffer or 100 mM pH 5.8 phosphate buffer. Leaching was then tested after two and ten days.

Stability. The stability of the material was investigated in milli-q water, 3.5 % NaCl, 100 mM pH 8 phosphate buffer or 100 mM pH 5.8 phosphate buffer, by checking the consistency of the measured fluorescence lifetime. About 0.05 g of material was dispersed in 5 mL of solution and tumbled for one month. After 0, 11, 17 and 33 days, the material and solution was separated, and the material re-dispersed in new solutions prior to measuring the fluorescence lifetime. This was done to avoid any interference of leached acridine, and also to avoid effects of solution pH changes that may occur when silica tumbles in water for a long period of time.

Fluorescence lifetime. In obtaining the fluorescence lifetimes of the immobilized acridine, fluorescence excitation was stimulated using subnanosecond pulses from a PicoQuant PLS light emitting diode with emission centered at 380 nm. The resulting fluorescence was collected using a PicoQuant PMA 175 photomultiplier, fitted with a 45 nm spectral filter centered at 452 nm. The photomultiplier response was digitized using a TimeHarp 260 Nano. The fluorescence lifetime values reported were determined by fitting exponential curves to the resulting fluorescence decay data using the FluoFit Pro software package.

^{29}Si MAS NMR and TEM characterization

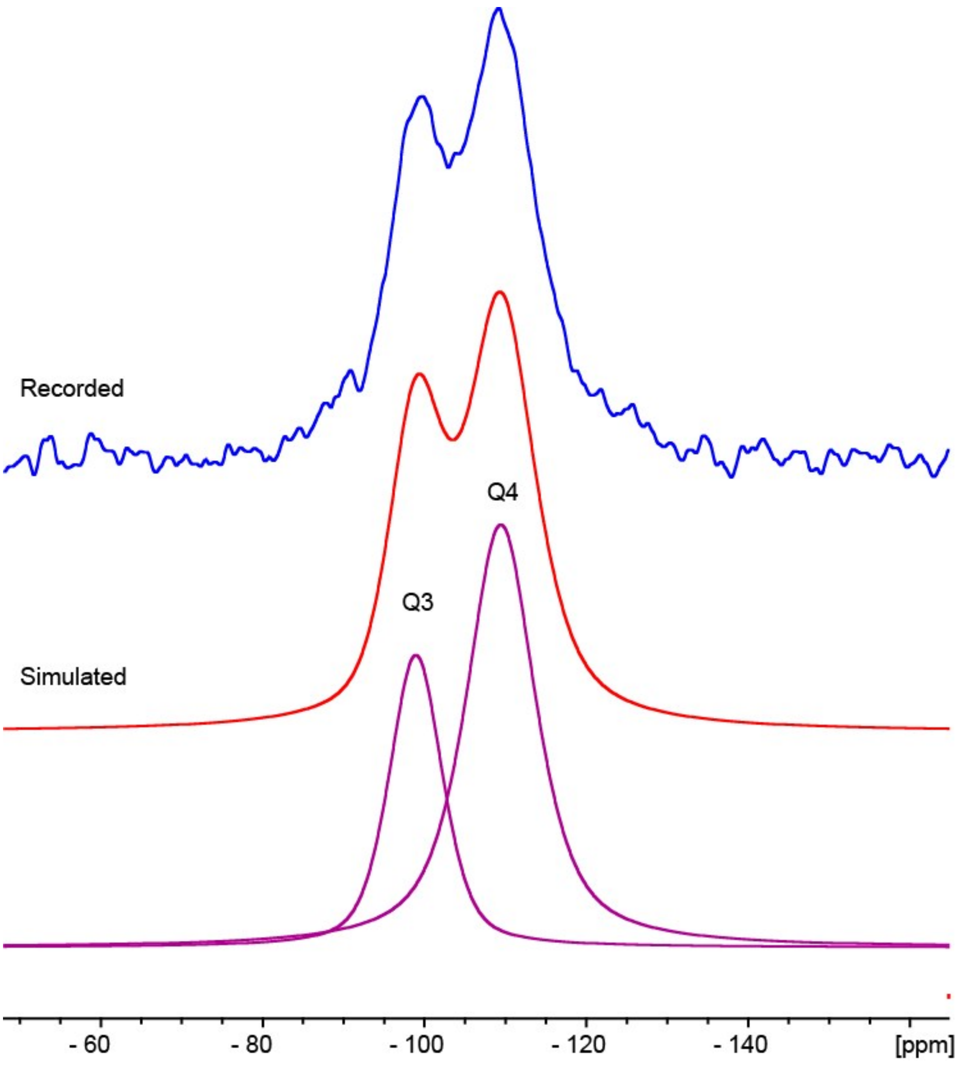

Figure S1. Recorded and simulated ^{29}Si Magic Angle Spinning (MAS) spectrum of the material. MAS spinning rate is 10 kHz, and 1000 transients were recorded with a relaxation delay of 120 seconds between each transient. The simulations (Topspin 3.2 software) were used to estimate the Q3/Q4 ratio of 0.52.

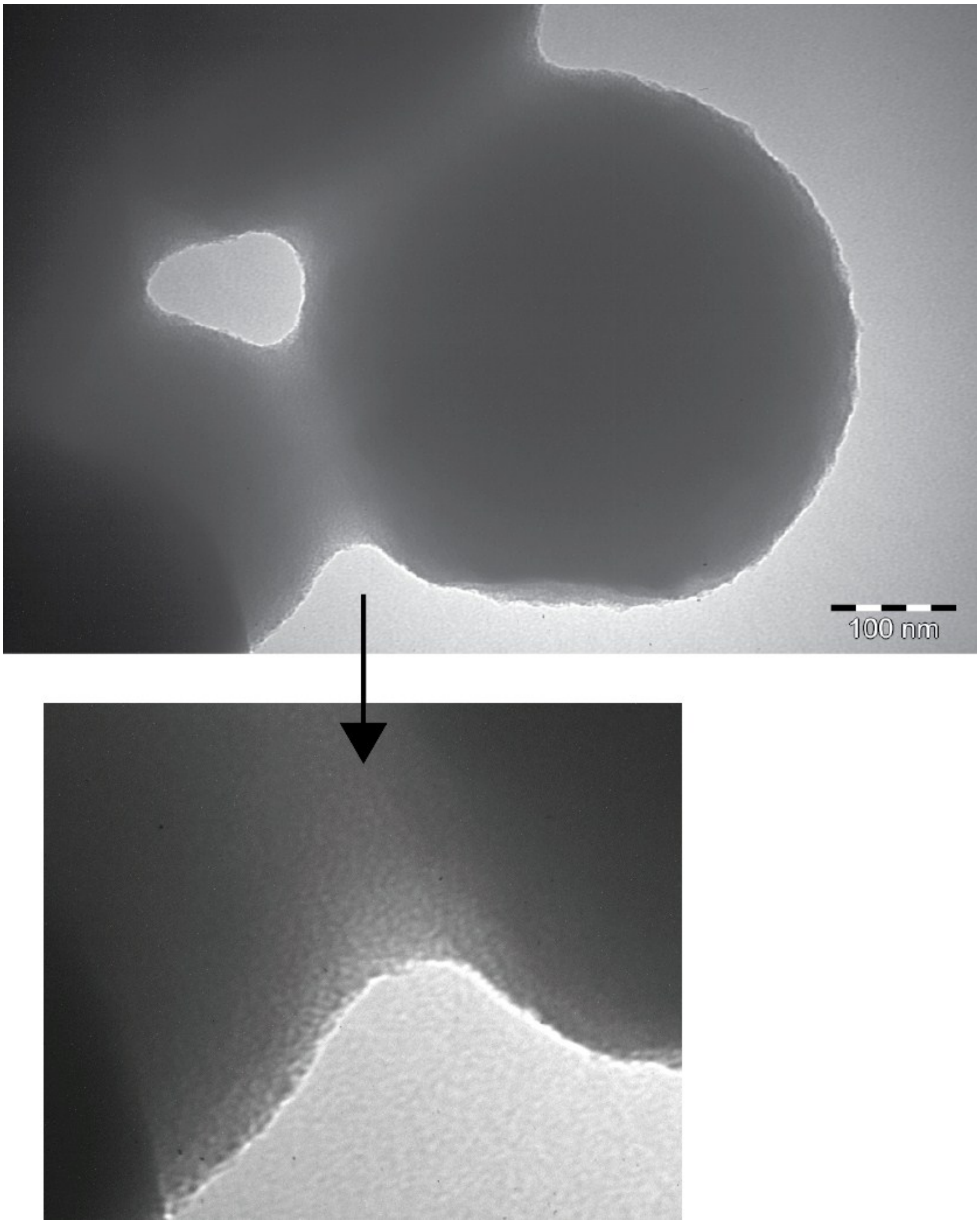

Figure S2. TEM micrograph at 250 000x of the mesoporous silica with the TTAB surfactant template intact. The porous network can be seen at the edges of the particles.

Leaching

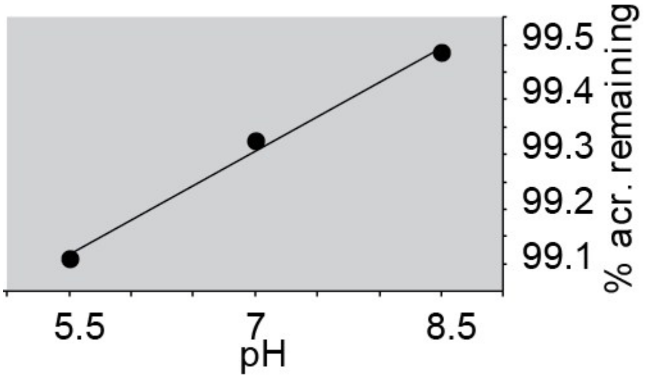

Figure S3. The leaching of acridine from the mesoporous silica after tumbling in 100 mM Carmody buffers of different pH for five days. The percentage of acridine remaining ($\%_{acr}$) after five days is linearly dependent at these pH values, and can be estimated from: $\%_{acr}$=0.13xpH+98.4.

Fluorescence spectra

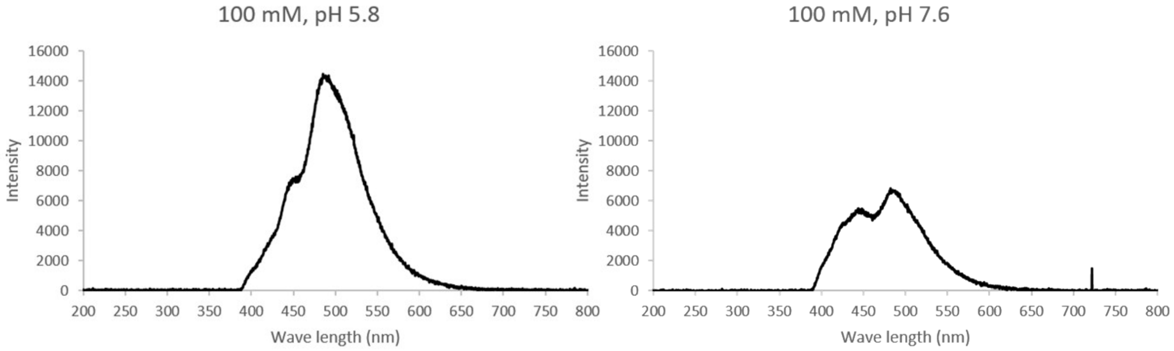

Figure S4. Fluorescence spectra of an equal amount of the material suspended in 100 mM phosphate buffers at pH 5.8 and 7.6.
